# Minor Variants of Orf1a, p33, and p23 Genes of VT Strain Citrus Tristeza Virus Isolates Show Symptomless Reactions on Sour Orange and Prevent Superinfection of Severe VT Isolates

**DOI:** 10.3390/v15102037

**Published:** 2023-09-30

**Authors:** Grazia Licciardello, Giuseppe Scuderi, Marcella Russo, Marina Bazzano, Moshe Bar-Joseph, Antonino F. Catara

**Affiliations:** 1 CREA—Council for Agricultural Research and Economics, Research Centre for Olive, Citrus and Tree Fruit, 95024 Acireale, Italy; 2 Agrobiotech Soc. Coop. z.i. Blocco Palma I, Stradale Lancia 57, 95121 Catania, Italy; gscuderi@agrobiotech.it (G.S.); mrusso@agrobiotech.it (M.R.); mbazzano@agrobiotech.it (M.B.); 3 The S. Tolkowsky Laboratory, Department of Plant Pathology, The Volcani Center, Agricultural Research Organization, Bet Dagan 50250, Israel; mbjoseph@gmail.com; 4 Formerly, Department of Phytosanitary Science and Technologies, University of Catania, 95123 Catania, Italy; antoninocatara@virgilio.it

**Keywords:** cross-protection, superinfection exclusion, genome sequencing, quick decline

## Abstract

The control of tristeza quick decline (QD) of citrus is based on the use of rootstocks that are tolerant or resistant to the Citrus tristeza virus (CTV), but some of them show bio-agronomic limits. The application of cross-protection (CP) has been insufficiently explored. The present study examined the possibility of QD control by cross-protection (CP) following reports showing the dependence of the CP strategy on the close genetic relationships between the protective and challenging CTV isolates. Taking advantage of deep sequencing technologies, we located six naturally infected trees harboring no-seedling yellow (no-SY) and no QD decline (mild) VT isolates and used these for challenge inoculation with three QD VT isolates. Symptom monitoring showed that all six Sicilian mild no-SY isolates, based on their genomic relatedness and mild symptoms reactions, provide effective protection against the three severe local VT isolates. The differences between the six mild and three severe isolates were confined to just a few nucleotide variations conserved in eight positions of three CTV genes (p23, p33, and Orf1a). These results confirm that the superinfection exclusion (SIE mechanism) depends on close genetic relatedness between the protective and challenging severe VT strain isolates. Ten years of investigation suggest that CP could turn into an efficient strategy to contain CTV QD infections of sweet orange trees on SO rootstock.

## 1. Introduction

The Citrus tristeza virus (CTV) is a long, flexuous, and filamentous Closterovirus, of the family Closteroviridae [1], transmitted semi-persistently by a few aphid species. The virus consists of a large (c. 19.3 Kb) +ssRNA genome, coding for twelve open reading frames (ORFs), flanked by 5′- and 3′- untranslated regions (UTRs) [2].

**Figure 1:**
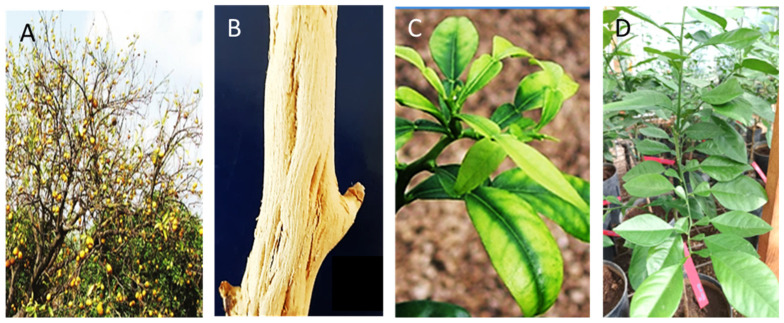
Different phenotypes induced by CTV-VT isolates used in this study. (**A**) Symptoms of decline on sweet orange grafted onto sour orange; (**B**) stem pitting on alemow; (**C**) seedling yellow on SO; (**D**) healthy-looking SO infected with no-SY isolates.

The CTV causes quick decline (QD) and stem pitting (SP) diseases (Figure 1). The main manifestation of QD is a sudden decline and the eventual death of citrus scions grafted onto sour orange (*Citrus aurantium*) rootstock (SO), whereas stem pitting (SP) affects sweet orange (*C. sinensis*), grapefruit (*C. paradisi*), lime (*C. aurantifolia*), alemow (*C. macrophylla*), and some other citrus varieties regardless of the rootstock. A third manifestation of CTV, seedling yellows (SY) (Figure 1), first described by Fraser [3] in Australia, is observed in inoculation of SO, lemon, and grapefruit in greenhouses [4,5].

In certain geographic areas where the *Aphis* (*Toxoptera*) *citricidus* is the main vector, field trees usually contain complex mixtures of different strains (genotypes) and their recombinants [6], but the mechanism by which they interact in plants has not been fully explored. In other areas lacking the efficient *A. citricidus* as a vector, such as Italy and Israel, CTV infections are mostly restricted to just a single genotype. Fu et al. [7] observed that the simultaneous inoculation of a mild and a severe CTV strain from different genotypes did not affect the other’s establishment in sweet orange, but the severe strain dominated.

In Sicily, where CTV is spread by *A. gossypii* and SO is still the most relevant rootstock, more than thirty thousand hectares of sweet orange are affected. The genetic structure of the local population of the virus includes three genotypes [8,9,10]. While the VT genotype is increasing (65%), T30, a mostly mild phenotype, is about 30%, only 4% are mixed, and the T36 genotype is sporadic. The VT decline isolates already caused the loss of 5 million trees, which have been replaced with plants on CTV-resistant rootstocks [11].

Alternative rootstocks that are tolerant to CTV show some horticultural problems due to the inadaptability of the citrange group’s rootstocks to calcareous and heavy soils and their susceptibility to soil fungal pathogens. Therefore, to continue the use of SO in areas with these soil types or where severe VT strains are present, there is major interest in finding cross-protective isolates that are suitable to prevent QD.

The mitigation of SP infection via cross-protection (CP) through the mechanism of superinfection exclusion has been achieved in countries where the disease is widespread. It started in Brazil in the 1960s [12], followed by South Africa [13,14], Perú [15], and Japan [16]. The same selection procedure, based on visual symptoms, has not successfully counteracted QD [17,18].

Research in Dawson’s lab by Folimonova et al. [19], using an infectious cDNA clone of a T36 isolate of CTV, demonstrated that the superinfection exclusion (SIE) mechanism operates only among isolates belonging to the same CTV genotype. Later studies showed that the p33 gene was involved in the SIE process [20,21,22] but was not fully elucidated in terms of genetic determinants and mode of action [23]. The relative fitness between the protective and challenge isolates has a role in this phenomenon [24]. A similar SIE mechanism was observed in our studies on no-SY VT isolates collected in the field, which prevented the infection of homologous SY VT isolates in SO seedlings [25], at that time related to mutations of the p23 gene, a determinant of the asymptomatic phenotype, which was previously described only in the T36 strain [26]. Recently, in South Africa SP isolates that are protective on grapefruit in field trials [27], minor nucleotide changes were revealed between mild and severe SP isolates of the T68 strain [28]. The present paper is based on these findings that indicated that CP works if both the protective and the challenging CTV isolates belong to the same strain.

In Sicily, two *C. macrophylla* plants infected with mild CTV isolates belonging to the VT strain were surprisingly found in a nursery. The SO plants infected with these isolates were symptomless and were found to be protected from superinfection seven months later from the SY, inducing a severe SG29 isolate [25].

The evidence shows continuity not only in the absence of symptoms of plants inoculated with no-SY isolates, but also high protective efficiency when challenged with aggressive variants. On the contrary, no protection was obtained when the T30 mild isolate was superinfected with the VT SY isolate.

Here, we report on (i) the selection of additional naturally occurring VT mild (no-SY) isolates that were totally asymptomatic on SO seedlings; (ii) the genome sequencing of newly identified isolates and comparison with other no-SY (mild) and SY (severe) isolates previously identified in the same area; (iii) the ability of the no-SY isolates to protect SO seedlings and sweet orange (SwO) grafted plants against VT-SY isolates for prolonged periods of symptom monitoring and ELISA tests.

The results show that eight variations in the sequences of three CTV genes, Orf1a, p33, and p23, shared by all the mild VT isolates we examined, allow these isolates to protect against severe VT isolates causing decline and seedling yellows, suggesting a role of one or more of these mutations in their function as disease elicitors that can perform SIE. Biological tests on SO, as a seedling or rootstock, showed the clear prevention of SY infection in plants protected by the homologous no-SY VT isolate.

## 2. Materials and Methods

### 2.1. Selection of Source Plants

Ten SY and no-SY isolates belonging to the VT strain were selected for this study, based on the results of large surveys in the citrus area of eastern Sicily carried out first using immunoenzymatic tests (ELISA), followed by real-time RT-PCR and biological tests on SO (Table 1). All the sites were located 10 to 30 km from the orchards first reported to show CTV-induced quick decline. Nine of these isolates, six VT no-SY and three VT SY, were used in the cross-protection tests. P3R1 was on a 30-year-old tree of Tarocco S. Alfio on SO close to Scordia. P7 (3C and 4C) was obtained through the bark inoculation of SG29 [25] in Pineapple sweet orange on Etrog citron. Tapi was a propagation of a Tarocco sweet orange grafted onto Carrizo citrange. Three no-SY isolates (Mac39, Mac101, and M55) infected *C. macrophylla* seedlings in a nursery; M39D was a Duncan grapefruit on citrange Carrizo inoculated with Mac39; Q7 originated from a Tarocco Lempso sweet orange in Palazzelli (Scordia); and Nan1 and Nan2 were Moro sweet oranges identified in the Lentini area.

### 2.2. Selection of No-SY Isolates

Source plants found to be affected solely by a VT genotype were further tested using bark graft inoculation (two pieces) of sour orange (SO), Mexican lime, and Duncan grapefruit seedlings (three replicates) in a temperature-controlled greenhouse (18–30 °C). The isolates failing to induce SY symptoms and those with SY were propagated on alemow or citrange (*Poncirus trifoliata x C. sinensis*) seedlings, which were continuously maintained in an insect-proof greenhouse.

### 2.3. Molecular Assessment of Genotype

Leaves or young shoots were used for CTV testing through ELISA by using the Reagent Set for Citrus tristeza virus (CTV) (Agdia, Inc., Elkhart, IN, USA) according to the manufacturer’s instructions. Samples showing positive reactions were eluted directly from the plate, washed with a phosphate-buffered saline (PBS) solution, tapped dry, and immediately processed or stored at −20 °C for at least 3–4 weeks and up to 4 months [9,29]. The positive reacting wells were filled with 50 µL of virus-release buffer (VRB) (10 mM Tris–HCl, pH 8.0 with 1.0% (*v*/*v*) Triton X-100), covered to prevent evaporation, and incubated by shaking them for 5 min at 65 °C. The resulting extracts were decanted and stored at 5 °C if processed on the same day or frozen at −80 °C for long-term storage. For real-time RT-PCR characterization, 5 µL were used as a template, using primers and probes described by Ruiz-Ruiz et al. [30], and amplified in a Rotorgene (Qiagen, Milan, Italy) apparatus.

### 2.4. Biological Stability of Protective Isolates Mac39 and Mac101 in Different Hosts

To evaluate the stability of the asymptomatic and protective phenotype, the no-SY isolates Mac39, M39D and Mac101, investigated for some years, were bud-propagated in greenhouses on SO and alemow seedlings and on Duncan grapefruit and Hamlin sweet orange grafted onto SO or alemow rootstocks. The plants thus inoculated were monitored for nine years for symptoms, with ELISA used to compare titer concentration and with molecular tests used to check the stability of the genotype. Moreover, the replication of no-SY and SY isolates was investigated two years after the superinfection, via the back-inoculation of tissues from treated plants on young SO seedlings, and monitored visually and through ELISA.

### 2.5. Long-Term Assays on Sour Orange and Sweet Orange on SO and Alemow

Cross-protection trials were carried out on SO seedlings, Hamlin, and Tarocco sweet orange plants grafted onto SO or alemow through the primary inoculation of no-SY CTV-VT isolates and superinfection with homologous severe SY isolates (Table 2). Long-term assays started in October 2014 in the greenhouse through the inoculation of one-year-old SO seedlings with Mac39 (two bark pieces) superinfected with SY P7/4C (February 2021) and M39D left without superinfection. A parallel trial was carried out by bud-grafting Hamlin SwO infected with Mac39 (two pieces) onto alemow seedlings and buds infected with Mac101 on SO. After six years of visual inspection and ELISA tests, six plants on alemow and six on SO were superinfected with the isolate P3R1, and the same number of plants were left protected. Each treatment included an adequate number of plants inoculated with only SY isolate and some healthy controls.

In autumn 2020, plants on SO were transferred to large pots (35L) outside the greenhouse and later into 50 L pots. In March 2023, plants were graft-inoculated in SO seedlings, with two replicas for each plant, monitored visually and through ELISA (Supplementary Table S1). The SO seedlings that were bark graft-inoculated with a severe isolate (Tapi) were used as controls.

### 2.6. Short-Term Tests of Cross-Protection Trials

Short-term tests simulating citrus nursery procedures were carried out in June 2020 and 2021 (Table 2). A large trial tested the isolates M39D and Mac101 against P7/3C on two groups of 16 ten-month-old SO seedlings. Each isolate included four blocks of four plants: (i) inoculated with no-SY and superinfected with P7/3C, (ii) only protected, (iii) inoculated with only SY, and (iv) healthy (without inoculation).

Other trials aimed to evaluate the time between primary CP inoculation and superinfection and to check the action of the Q7, M55, and Nan1 no-SY isolates selected in the field. The SO seedlings and sweet oranges on SO were bark graft-inoculated (two pieces) with no-SY isolates and superinfected, 3 to 15 months later, with different SY isolates (P7/3C, P3R1, and Tapi) (Table 2). Control plants were left just protected or healthy and not inoculated at all. Few plants were inoculated only with SY isolates since the plants started to decline and died after no more than 10 months. Monitoring was performed visually and through ELISA (Supplementary Table S1).

### 2.7. High-Throughput Genome Sequencing

The genomes of four CTV isolates were elucidated through the deep sequencing of small RNAs from 100 mg of bark and leaf tissues of SO (M55 and N1), grapefruit (M39D), and sweet orange grafted on SO (Q7) using the MirPremier MicroRNA isolation kit (Sigma, Kawasaki, Japan). Library construction of nucleic acids was carried out using the NEXTflex Small RNA Sequencing kit (Bio Scientific, Avondale, AZ, USA). The service providers were requested to provide 20 million read pairs per sample from Illumina sequencing. The small RNA library was then multiplexed, clustered, and sequenced on an Illumina HiSeq 2000 (TruSeq v3 chemistry) with a single-read 50-cycle sequencing protocol, plus indexing following the previously described procedure [11]. The sequencing run was analyzed with the Illumina CASAVA pipeline (v1.8.2), with demultiplexing based on sample-specific barcodes. Small RNA adapters were removed using the “Trim sequences” option of the CLC Genomics Workbench (v 6.0.4).

### 2.8. Sequencing of p33 and p23 PCR Amplicons

The full lengths of the p33 and p23 genes were sequenced through Sanger in ten samples consisting of SY and no-SY isolates artificially inoculated on different plant sources. Total RNA was extracted from 100 mg of fresh, fully expanded citrus leaves, previously pulverized with liquid nitrogen using the Tri Reagent^®^ (Sigma Aldrich, St Louis, MO, USA) according to the manufacturer’s instructions. The p23 gene was amplified via RT-PCR using the primers p23extFw (GGTTGTATTAACTAACTTTAATTCG) and p23extRew (AACTTATTCCGTCCACTTCAATCAG) (709 bp amplicon). RT-PCR was performed in a single closed tube containing a final volume of 20 μL using qScript XLT 1-Step RT-PCR Kit (Quantabio, Beverly, MA, USA) with the following mixture: 1X One-Step HiFi PCR ToughMix, 1X One-Step RT master mix, and 0.2 μM primers. The reaction mix was incubated in a Flexigene FFG02FSD Benchtop Thermal Cycler PCR (Techne, Midland, ON, Canada) at 50 °C for 15 min, 94 °C for 5 min, 40 cycles of 94 °C for 30 s, 56 °C for 30 s, 72 °C for 1 min, and a final extension step at 72 °C for 7 min for p23 gene amplification. For p33 amplification primers, p33 forward (5′ GATGTTTCCTTCGCGAGC 3′) and p33 reverse (5′ CCCGTTTAAACAGAGCAAACGG 3′) (983 bp amplicon) were used [31]. PCR amplification conditions were as follows: denaturation at 50 °C for 15 min, 94 °C for 5 min, 35 cycles of 92 °C for 30 s, 65 °C for 45 s, 68 °C for 1 min, and a final extension at 68 °C for 10 min. PCR products that were shown to be associated with single bands on an agarose gel were enzymatically purified through ExoSAP-IT™ PCR Product Cleanup Reagent (Thermo Fisher Scientific, Milan, Italy) following the instructions. Purified amplicons were sent for direct sequencing in both directions to Eurofins Genomics (Milan, Italy).

### 2.9. Bioinformatics Analysis

Unpaired reads without adapter contaminants were indexed using Bowtie2-build program version 2.1.0., and alignment qualities were assessed using Qualimap [32]. Quality trimmed data were imported into Bowtie2 v. 2.1.0, and a preliminary plant host filtering step was performed after alignment with the chromosomes of *Citrus sinensis* (NC_023046 to NC_023054), chloroplast (NC_008334), and miRNAs (retrieved from MiRBase database) using the default parameters. For CTV genotype detection, unmapped reads were aligned using a reference sequence-guided assembly approach [25] to six reference genomes representative of the six main CTV genotypes [33] (VT: KC748392, B165: EU076703, T3: EU857538, T36: EU937521, RB: FJ525435, and T30: KC748391) plus the VT no-SY isolate Mac39 (KJ790175). The consensus sequences generated using SAM tools 0.1.19 were deposited in the Genbank database under the accession numbers OR387854 (M39D), OM803129 (Q7), OP345182 (Nan1), and OP345183 (M55) and analyzed for similarity against sequences of other previously studied isolates [11,25]. The mapping alignments were further measured using three metrics generated by Qualimap [32] analysis: read counts, percentage of reads count, and percentage of genome fraction coverage (GFC) at 50X. Graphical representations showing the quality of reads alignment against each reference genome, generated using Qualimap, were assessed for a fast evaluation of the unequivocal presence of the virus or viroid in a library. Multiple sequence alignments, a distance matrix, and a phylogenetic analysis were performed using MEGA11 [34] and Geneious [35]. RNA secondary structure prediction was performed using the RNAfold web server (accessible at http://rna.tbi.univie.ac.at/cgi-bin/RNAWebSuite/RNAfold.cgi, accessed on 5 July 2023).

## 3. Results

### 3.1. Selection of No-SY Source Tree Isolates Spreading in Different Areas of Sicily

After a wide survey in the citrus-cultivated area of eastern Sicily, considering citrus species of diverse age, sites, and type of cultivations, we selected a total of six source trees affected by VT no-SY (mild) isolates. Three were selected among a group of 19 two-year-old alemow seedlings that showed typical CTV symptoms of vein clearing and stem pitting, but were asymptomatic when inoculated on SO seedlings. Two of the isolates (Mac39 and Mac101) were already described [25]. Isolate M55 was obtained from the same group of naturally infected alemow seedlings plants, and it showed similar mild symptoms on alemow and grapefruit (small pits on the woods). The other isolates originated from sweet orange trees on SO growing regularly in citrus orchards, without any symptom of systemic diseases (Table 1).

### 3.2. Long-Term Trials Show No-SY VT Isolates Induce Durable Cross-Protection

All the propagation progeny of Mac39 and Mac101 obtained through bud or bark grafting of grapefruit, sweet orange, alemow, and SO did not induce symptomatic reaction in host plants for years in greenhouse but showed SP on grapefruit and alemow when transferred outside. The capability to counteract superinfection through the SY isolate was maintained in the greenhouse and in the field. The subsequent back-inoculation of SO seedlings, via bark tissue taken from plants protected with no-SY isolates nine years before (October 2014) and superinfected a few months later, did not differ in growth from the uninoculated control, confirming the stability of the asymptomatic and cross-protective phenotype. The SO plants inoculated only with the SY isolate started to turn yellowish after two months and declined after one year.

The Hamlin plants on SO, cross-protected with Mac39 in October 2014 and superinfected a few months later with the SY P7/4C isolate, developed regularly after six years in greenhouse and are still growing three years later in large pots outside the greenhouse. The SO seedlings, which were cross-protected and non-superinfected, do not show any symptoms. No SP was observed on Hamlin and SO. The plants that were not protected showed a reduced growth.

Hamlin sweet orange plants infected by cross-protective Mac39 or Mac101 isolates, propagated on SO and on alemow, developed regularly in a greenhouse for six years without any relevant symptoms. Subsequent superinfection with the severe SY isolate P3R1 did not induce any suspicious modifications 22 months post-inoculation. The alemow did not show stem pitting in the greenhouse.

Immunoenzymatic tests randomly carried out on plants that were cross-protected and superinfected, or not protected, showed that the average OD value was very similar, regardless of the isolate (Supplementary Table S1). To ascertain if the SY isolate used for superinfection was present anyway, several plants that were protected and superinfected were re-tested on SO seedlings using bark graft-inoculation. Five months later, the seedlings did not show any SY symptoms and grew generously, despite the infection of the CTV VT isolate as ascertained by ELISA and molecular analysis, leading to the assumption that the replication of the superinfecting SY isolates was blocked.

### 3.3. Short-Term Trials Confirm Cross-Protection in Different Combinations

**Figure 2:**
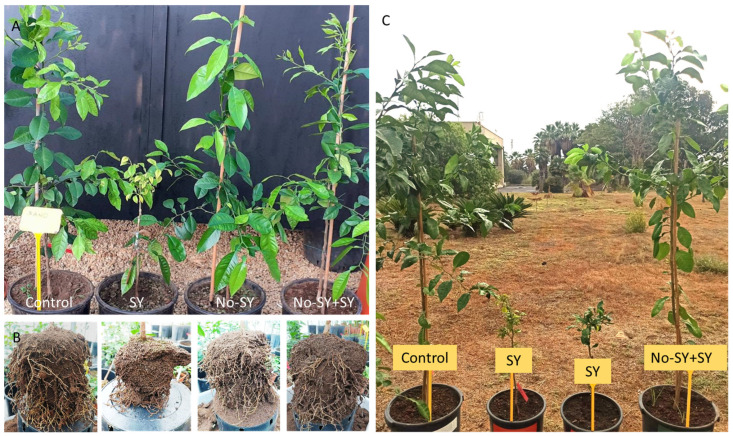
Different growth and root development of SO seedlings inoculated with CTV SY, no-SY, and no-SY + SY isolates in comparison to healthy control, 8 (**A**,**B**) and 15 (**C**) months after the challenge inoculation. Pre-inoculation with no-SY isolates was performed eleven months before.

The main short-term trial tested two no-SY isolates (M39D and Mac101) against the severe SY isolate P7/3C on SO seedlings, differently treated: (i) protected with no-SY and superinfected, (ii) only protected, (iii) inoculated with only SY, and (iv) healthy. Six months after inoculation with P7/3C, the SO plants showed foliar yellowing symptoms and a remarkable reduction in their root system, while the no-SY Mac39 and Mac101 plants, and plants that were cross-protected and later challenged with SY P7, remained asymptomatic (Figure 2).

Primary CP inoculation and superinfection was also tested on Tarocco sweet orange that was grafted on SO pre-inoculated with Q7 and superinfected with P7/3C after 15 months. The protected plants did not show any symptoms, whereas those not protected showed a pale foliage. A similar result was obtained when Hamlin sweet orange was protected with M39D and superinfected with P7/3C three months later. Additional trials carried out on SO seedlings protected with two no-SY isolates (M55 and Nan1) in June and superinfected in September or October with Tapi or P3R1 did not show any symptom (Table 2), while seedlings not protected and infected with SY isolates showed a yellowing of foliage after 5–6 months and a decline approximately one year after the inoculation.

### 3.4. Genome Analysis Reveals No-SY Isolates Are Nucleotide Variants of the SY SG29 Isolate

All the libraries analyzed in this study generated a variable number of single reads ranging from 11 M to 51 M (Table 3). After the host filtering step, unmapped reads were analyzed for the presence of CTV to reconstruct the full genome sequence. The results showed that the samples were infected by a VT strain, as revealed by the high number of reads mapped and the 100% coverage with the VT reference genomes [11,36] (Table 3), as well as by the graphical representations generated using Qualimap. The genomes were 19,250 nt in length and structurally identical to known CTV isolates, with 12 open reading frames (ORFs) and including 107 nt in the 5′UTR and 272 in the 3′ UTR.

**Figure 3:**
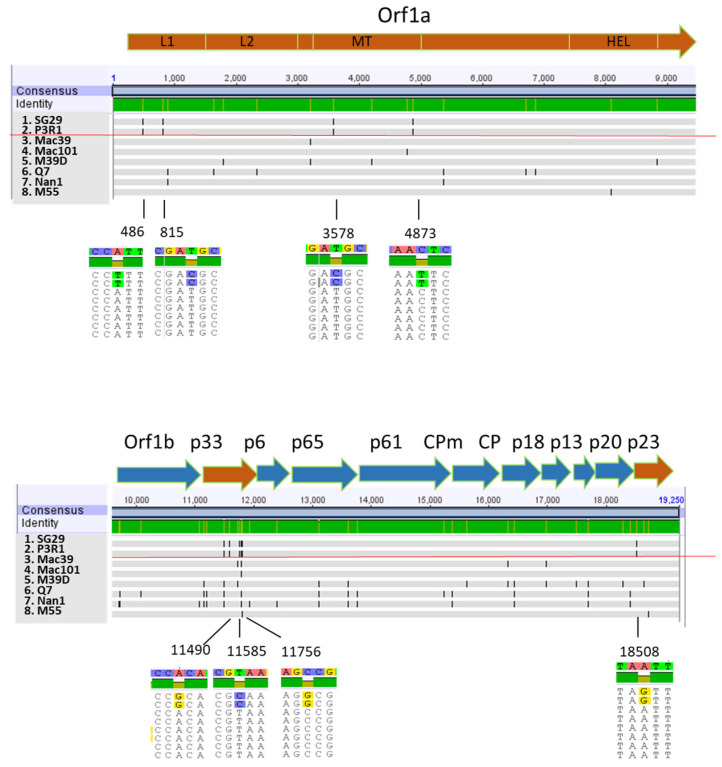
Comparative nucleotide genomic differences among the Citrus tristeza virus SY and no-SY isolates used in this study. The position of polymorphisms differentiating SY and no-SY isolates is shown corresponding to the Orf1a, p33, and p23 genes (orange arrows). L1 and L2, papain-like leader proteases; MT, methyltransferase; HEL, helicase; CPm, minor coat protein; CP, coat protein.

Comparative genome analysis showed that Q7, M55, Nan1, and M39D share 99% of nucleotide homology with the previously sequenced SG29 (KC748392) and P3R1 isolates [11,37] that induced SY, as well as with Mac39 (KJ790175) and Mac101 (MW689620), two previously described no-SY isolates [25]. The results confirm that the no-SY isolates are sequence variants of the SG29 isolate. Multiple alignments, including two SY (SG29 and P3R1) and six no-SY isolates (Mac39, Mac101, M39D, Q7, M55, and Nan1), revealed the presence of a total of 63 out of 19,250 polymorphisms spread along the entire genome among the isolates. It was interesting to note that eight polymorphisms positioned in the Orf1a, p33, and p23 genes were firmly fixed in SY and no-SY isolates (Figure 3; Table 4). Orf1a polymorphisms were located within the N-terminal domain of the L1 leader protease domain (L1NTD) (486 nt and 815 nt) and the methyltransferase domain (3578 nt and 4853 nt), whereas no changes were detected in the L2 protease domain. Six variations are nonsynonymous mutations that induce amino acid changes (Table 4). No changes in the RNA secondary structure prediction or in amino acid polarity were detected.

According to the number of polymorphisms, the Q7 isolate revealed the highest distance from SG29 and P3R1, with an identity level of 99.77%, differing for 31 nucleotides, followed by M39D and N1 (99.80% and 99.81%, respectively), with 30 and 29 nucleotide differences, respectively (Figure 3). These three isolates originated from field sweet oranges (Q7 and N1) and grapefruit that was artificially inoculated with Mac39 in greenhouse conditions (M39D).

**Figure 4:**
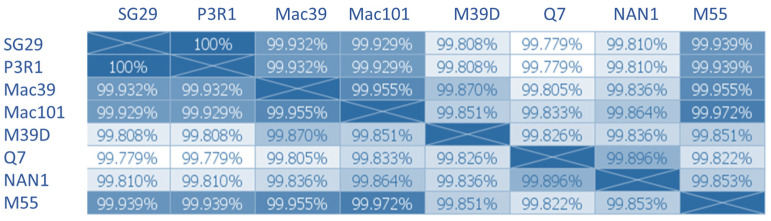
Distance matrix showing the genetic distance between the genomes of eight Citrus tristeza virus SY and no-SY isolates belonging to the VT strain.

On the contrary, isolates Mac39, M39D, Mac101, and M55, which originated from *C. macrophylla* seedlings, showed the highest distance from SG29 and P3R1, with 99.93% sequence identity and only 20 nt, 18 nt, and 18 nt differences, respectively (Figure 4).

### 3.5. The Nucleotide Variations on the p33 and p23 Genes Are Conserved in All the No-SY Isolates

To confirm the nucleotide variations detected in the no-SY isolates through HTS analysis, we sequenced the entire p23 and p33 genes of seven isolates (Mac39, M39.12, M39D, Mac101, M55, Nan1, and Q7) from plant sources grown in a greenhouse (Table 5). Moreover, two additional SY isolates were analyzed: P7/3C derived from the inoculation of SG29 on Pineapple sweet orange, and Nan2, a field tree growing close to Nan1.

A nucleotide sequence analysis of the p23 gene revealed that all the no-SY isolates contain an adenine at position 161 and maintain this polymorphism in all the host plants analyzed. On the contrary, the P7/3C and Nan2 SY isolates contain a guanine in the same position (Table 5), confirming the HTS results obtained for the SG29 and P3R1 isolates.

The positions 630 nt, 725 nt, and 896 nt of the p33 gene (A, T, and C, respectively) differed between the no-SY and SY isolates according to HTS sequencing results (Table 5). All the no-SY protective isolates also confirmed the maintenance of the polymorphisms in the additional samples analyzed using Sanger sequencing, regardless of the host. The Mac39 and Mac101 isolates maintained the same sequence after the passage from alemow to grapefruit and the number of transmissions. However, the presence of these nucleotides was also detected in the P7/3C and Nan2 SY isolates differently from the SG29 and P3R1 sequenced using HTS. Although unexpected, this result suggests that mutations on the p33 gene are not strictly linked to pathogenicity or that such a range of genetic changes is not expected to happen immediately. It is evident that the G–A mutation of p23 abolishes the CTV SY reaction in SO plants, while the mutations of p33 allow the A-possessing p23 mutant to become dominant in an SY-sensitive citrus host.

## 4. Discussion

The development of CTV-related disease syndromes such as quick decline, stem pitting, or seedling yellow depends on the infecting virus isolates, as well as on the citrus rootstock and scion combination [6]. Cross-protection against the manifestation of CTV stem pitting has been successfully practiced throughout the world’s largest citrus industries on a large scale and for long periods [13]. Similar attempts to protect citrus grafted on SO rootstock against the CTV quick decline (QD) problem have so far failed to become practical.

Recent research indicates that the proper selection of CTV isolates that are suitable for providing superinfection interference (SIE), the basic term of practical cross-protection, relies mainly on close genetic identities between the protective and the challenging isolates [19,20,27,38].

Previously, we reported that just a few nucleotide changes observed in the CTV-VT isolates Mac39 and Mac101 rendered these symptomless, compared to SG29, the SY-inducing CTV-VT isolate from Sicily [25]. In the present study, we located four additional no-SY isolates (M39D, Q7, Nan1, M55) from different citrus source plants and different Sicilian geographies. The cross-protective abilities of these mild isolates were tested using challenge inoculation with three CTV-VT-SY isolates (P7/3C and/4C, P3R1, and Tapi). The protected plants remained symptomless seven years before the superinfection, two years after it in long-term trials, and for more than two years in the short-term.

The six mild isolates, selected based on their genetic homology base, were found to effectively cross-protect against the three severe VT SY isolates, supporting the notion that close genetic traits trigger the SIE mechanism [19,20,38]. The mechanism underlying the ability of the primary virus to be indifferent to the infection of distant genotypes, while specifically excluding infection by closely related virions, remains to be explained. However, the theory behind the ability of all mild CTV isolates to prevent superinfection of their closely related severe isolates indicates the presence of a “self-defense” mechanism during the replication process [39]. The origins probably stem from an existential necessity to evade the danger of quasi-species population collapse, resulting from the multiple errors of viral RNA replication [40]. Assuming such a scenario, only the closely related challenging isolates could mimic the natural “resident” RNA mutants for being cleared during replication process and allow the continued genetic uniformity of the population of the already adapted and dominating virus genotype.

The persistent lack of leaf yellowing and root decline in SO seedlings inoculated with VT no-SY isolates, which extended to more than nine years, confirms the stability of the asymptomatic phenotype associated with the p23 mutation [25]. Furthermore, the lack of any leaf yellowing and root deterioration among SO seedling plants inoculated with tissue from cross-protected plants confirms that the replication of the superinfected VT-SY isolate was blocked in plants protected by no-SY isolates.

Genomic analysis of the VT SY isolates SG29 (KC748392) and P3R1 [37] showed their similar identities, while a total of 63 nucleotide variations were detected between the two VT SY and the six no-SY isolates. The highest density of changes was mainly detected in the 5′-end of the genome, which was the most variable compared to the 3′-end [33]. Most of the firmly fixed nucleotide variations between the SY and no-SY isolates were detected in the p33 and the Orf1a genes, a condition similarly described for mild and severe SP isolates of the T68 genotype [28]. The observed polymorphisms are conserved in isolates originating from a nursery plot of alemow (Mac39, M39D, M55, and Mac101) and in the additional no-SY isolates Nan1 and Q7 from old citrus trees located in distant areas of Sicily. Since the aphid transmission of CTV in nursery plants is expected to be less complex than in the open field, the distant location of no-SY isolates leads to the hypothesis that they were spread by aphids [41].

The eight genetic changes differentiating mild and severe isolates were spread over three CTV genes Orf1a, p33, and p23, previously shown to have crucial interactions with their host plants [21,26]. The dominance of the mild isolate suggests a strong selection pressure toward newly mutated CTV-VT isolates lacking the effector capacity [18]. The small number of nucleotide replacements, with just one on the SY effector gene (p23), could be explained by the reported slow mutation of the CTV [42,43].

Regarding Orf1a, two nucleotide variations between the SY and no-SY isolates (one of them non-silent) are located in the N-terminal domain of the L1 leader protease domain (L1NTD), whereas no variations occurred in the L2 protease domain. The substitution of the L1-L2 leader proteases’ region with a cognate sequence from a heterologous virus isolate genome resulted in a loss of virus ability to exclude superinfection by the parental virus, indicating the involvement of this region in SIE [44,45]. However, this region is not sufficient for eliciting the exclusion, and homology with p33 or other viral factors are also necessary [45,46]. Thus, the minor sequence variations between VT SY and no-SY isolates, here reported in Orf1a in association with other modifications in p33 and p23 genes, have a role in preventing the superinfection of genetic close isolates.

The p33 gene is described as a viral effector that influences CTV pathogenicity by modulating a host immune response [21] through interaction with CP, p20, and p23 CTV proteins [22,46]. In T36, it acts as a determinant of SIE at the whole organism level but is not required for exclusion at the cellular level [47]. In our study, the consistent presence of the p33 mutations from different host plants, locations, and ages, confirms its biological role in the VT strain. The ‘ATC’ variation detected in the VT no-SY isolates suggests that the mutations on p33 allow the virions with the p23 and Orf1a mutations to change from SY to no-SY. However, the finding of similar p33 polymorphisms in P7 and Nan2 SY isolates, lacking the p23 mutation, suggests that the observed p33 mutations were not sufficient for the shift from SY to no-SY.

The p23 gene, which is reported to be involved in SY reactions in SO and grapefruit of the T36 CTV isolate [26], is confirmed to have an effect on the VT strains. The nucleotide variation of serine to asparagine at position 54 is confirmed in the additional isolates that were fully or partially sequenced in this study (Table 5). The substitution of Ser54 with Asn54 in the 50–54 region was reported to cause substantial changes in the phenotypic expression in specific hosts [48,49]. It could be that the G-to-A mutation of p23 abolishes the CTV SY reaction in SO plants, and the ‘ATC’ mutation of p33 allows the A-possessing mutant to spread and become dominant in an SY-reacting SO hosts.

The genetic relatedness of the CTV isolates described in this study and the cross-protection results are in accordance with previous studies showing the association of the SIE phenomenon with just a few viral determinants [23]. In *Potyvirus*, some effectors of SIE acting at the protein level and not at the RNA level have been described as being able to prevent the superinfection of a closely related strain or virus [23].

Advances in genetic analyses of virus genomes offer unprecedented opportunities for the rapid selection of closely related protective isolates; this is a major development compared with the lengthy biological assays that were employed in the past for locating protective isolates. In the past, there was a challenging opportunity to reverse the QD phase not only by cross-protection of previously uninfected citrus trees, but also by reversing the disease situation in trees already exhibiting QD. An area of research with considerable significance for citrus, where transgenic resistance was explored for other crop plants [23,50], was so far non-effective [51]. However, the application of cross-protection will have to be closely associated with the continuous genetic monitoring of the spreading CTV strains in the treated areas, since protection is only expected against the severe isolates of the VT strain.

## 5. Conclusions

The study describes the close genetic relationships between mild no-SY and severe SY CTV-VT isolates selected in Sicilian citrus groves and shows their long-term cross-protection interaction. Ten years of investigation on cross-protection suggest that CP becomes an efficient strategy to contain CTV QD infections in plants grafted on SO, in VT infested areas where the highly efficient CTV vector, *A. citricidus*, is still absent. The details of the SIE mechanism are still unknown but the considerable dependence on close genetic relationships between the protective and superinfecting (challenging) CTV isolates can be plausibly explained by the expected necessity of replicating RNA viruses to evade the danger of quasi-species population collapse resulting from the high mutation-prone process of viral RNA replication [40].

Beyond the aspects related to molecular mechanisms, which open new areas of investigation that are probably also useful for other virus diseases [23,50], this “recipe” may become a beneficial tool for the prevention of yield losses caused by the decline in SY isolates and the reduction in agro-chemical inputs, which are experienced by the SP isolates in many countries that have opted for cross-protection [39,52]. Ongoing field trials will help to explain some relevant ecological and biological aspects.

## Figures and Tables

**Table 1:** Biological and molecular characteristics of source trees infected by the Citrus tristeza virus SY and no-SY isolates used in this study.

Source Trees	Age (yrs)	Location	CTV Isolate	Genotype and Phenotype	Mode of Infection
Alemow seedlings	2	Citrus nursery, Belpasso	Mac39	VT no-SY	Aphid
			Mac101	VT no-SY	Aphid
			M55	VT no-SY	Aphid
Duncan Gpf/TC	2	Greenhouse, Catania	M39D	VT no-SY	Inoculation
Tarocco Lempso SwO/CC	20	Orchard, Lentini	Q7	VT no-SY	UNK
Moro SwO/SO	30	Orchard, Lentini	Nan1	VT no-SY	UNK
Moro SwO/SO	30	Orchard, Lentini	Nan2	VT SY	UNK
Pineapple SwO/CC	1	Greenhouse, Catania	P7(3C&4C)	VT SY	Inoculation
Tarocco S.Alfio SwO/SO	25	Orchard, Scordia	P3R1	VT SY	UNK
Tarocco Tapi SwO/SO	10	Greenhouse, Catania	Tapi	VT SY	Inoculation

Gpf/TC: grapefruit/Troyer citrange; Swo/CC: sweet orange/Carrizo citrange; SwO/SO: sweet orange/SO; UNK: unknown.

**Table 2:** Plan of cross-protection trials on sour orange seedlings and sweet orange/rootstocks combinations through the primary inoculation of Citrus tristeza virus VT no-SY isolates and superinfection with homologous severe SY isolates.

CP Isolate	SY Isolate	Host Plant	CP Inoculation	SY Inoculation	No. of Plants	
					CP	CP + SY
		**Long term trials**				
**M39D**	None	SO	October 2014	//	6	//
**Mac39**	P7/4C	SO	October 2014	February 2021	2	4
**Mac39**	P3R1	H SwO/Ale	October 2014	October 2021	4	6
**Mac101**	P3R1	H SwO/SO	October 2014	October 2021	2	4
		**Short term trials**				
**M39D**	P7/3C	SO	June 2020	May 2021	4	4
**Mac101**	P7/3C	SO	June 2020	May 2021	4	4
**Q7**	P7/3C	T SwO/SO	June 2020	September 2021	5	5
**M39D**	P7/3C	H SwO/SO	June 2020	August 2021	5	5
**M55**	Tapi	SO	June 2020	September 2021	2	4
**Nan1**	P3R1	SO	June 2020	September 2021	2	4
**Nan1**	Tapi	SO	June 2020	October 2021	2	4

CP: cross-protective; SY: SY isolate used for the super infection; SO: sour orange; H SwO/SO: Hamlin sweet orange on SO; T SwO/SO: Tarocco sweet orange on SO; H SwO/Ale: Hamlin sweet orange on alemow.

**Table 3:** HTS sequencing results of four CTV-VT no-SY isolates obtained in this study. The read counts and genome coverage, obtained after alignments of siRNA libraries with seven CTV reference genomes, are shown. Positive identifications are associated with genome coverage over 90%.

Sample	Reads Number	CTV Isolate Reference Genomes (Reads/Coverage)						
		VT KC748392	VT No-SY KJ790175	T30 KC748391	RB FJ525435	T36 EU937521	T3 EU857538	B165 EU076703
M39D	11.64 M	1.23 M *100%*	1.34 M *100%*	482.907 *75%*	376.301 *65%*	385.023 *55%*	1.06 M *80%*	1.05 M *80%*
Nan1	17.05 M	3.05 M *100%*	3.17 M *100%*	649.833 *74%*	526.440 *67%*	495.293 *60%*	1.28 M *85%*	1.32 M *85%*
Q7	22.73 M	2.24 M *100%*	2.56 M *100%*	762.004 *75%*	736.293 *65%*	561.395 *55%*	1.57 M *80%*	1.98 M *80%*
M55	51.32 M	2.82 M *100%*	2.81 M *100%*	761.194 *75%*	587.717 *65%*	546.631 *60%*	1.07 M *85%*	1.55 M *85%*

**Table 4:** Comparative nucleotide variations in the Orf1a, p33, and p23 genes of eight Citrus tristeza virus SY and no-SY isolates obtained from different citrus varieties’ source trees located in different places.

Isolate	GenBank ^1^	Phenotype	Orf1a ^3^				P33			P23
			486 nt *127 aa*	815 nt *236 aa*	3578 nt *1157 aa*	4873 nt *1589 aa*	11,490 nt *210 aa*	11,585 nt *242 aa*	11,756 nt *299 aa*	18,508 nt *54 aa*
**SG29**	KC748392	SY	T/Phe	C/Asp	C/Asp	T/Iso	G/Pro	C/Ala	G/Gly	G/Ser
**P3R1**	/ ^2^	SY	T/Phe	C/Asp	C/Asp	T/Iso	G/Pro	C/Ala	G/Gly	G/Ser
**Mac39**	KJ790175	No-SY	A/Ile	T/Asp	T/Asp	C/Thr	A/Pro	T/Val	A/Ala	A/Asn
**Mac101**	MW689620	No-SY	A/Ile	T/Asp	T/Asp	C/Thr	A/Pro	T/Val	A/Ala	A/Asn
**M39D**	OR387854	No-SY	A/Ile	T/Asp	T/Asp	C/Thr	A/Pro	T/Val	A/Ala	A/Asn
**M55**	OP345183	No-SY	A/Ile	T/Asp	T/Asp	C/Thr	A/Pro	T/Val	A/Ala	A/Asn
**Q7**	OM803129	No-SY	A/Ile	T/Asp	T/Asp	C/Thr	A/Pro	T/Val	A/Ala	A/Asn
**Nan1**	OP345182	No-SY	A/Ile	T/Asp	T/Asp	C/Thr	A/Pro	T/Val	A/Ala	A/Asn

^1^ Genbank accession numbers of the corresponding genome sequences. ^2^ The genome of P3R1 was not submitted because it is redundant with SG29. ^3^ Nucleotide position starting from the first base of the genome and amino acid position from the first of the corresponding translated protein.

**Table 5:** Nucleotide variations detected through HTS and the Sanger sequencing of the entire p33 and p23 of the SY and no-SY isolates infecting different hosts.

Isolates	Sequencing	Host	Phenotype	p33			p23
				630 nt (11,490 nt)	725 nt (11,585 nt)	896 nt (11,756 nt)	161 nt (18,508 nt)
SG29 *	HTS	SwO	SY	G	C	G	G
**P7**	Sanger	SwO	SY	A	T	C	G
**P3R1**	HTS	SwO	SY	G	C	G	G
**Nan2**	Sanger	SO	SY	A	T	C	G
**Mac39**	HTS	Alem	no-SY	A	T	C	A
	Sanger	Alem	no-SY	A	T	C	A
**M39.12**	Sanger	Alem	no-SY	A	T	C	A
**M39D**	HTS	Gpf	no-SY	A	T	C	A
	Sanger	Gpf	no-SY	A	T	C	A
**Mac101** **Mac10**	HTS	Alem	no-SY	A	T	C	A
	Sanger	Gpf	no-SY	A	T	C	A
**M55**	HTS	SO	no-SY	A	T	C	A
	Sanger	SO	no-SY	A	T	C	A
	Sanger	Citr	no-SY	A	T	C	A
**Q7**	HTS	SwO	no-SY	A	T	C	A
	Sanger	SwO	no-SY	A	T	C	A
**Nan1**	HTS	SO	no-SY	A	T	C	A
	Sanger	SO	no-SY	A	T	C	A

* Gray bands show two groups of isolates originating from the graft bark inoculation from the same source, shown in the top position of the section.

## Data Availability

All sequencing data obtained in this study were included in the manuscript and submitted to the GenBank database under the accession numbers OR387854, OM803129, OP345182, and OP345183.

## Supplementary Materials

The following supporting information can be downloaded at: https://www.mdpi.com/article/10.3390/v15102037/s1, Table S1: Comparative analysis of CTV detection on leaf samples taken from plants cross-protected and superinfected or not, used in the trials of CP reported in Table 2.

## References

[1] Martelli G.P., Abou Ghanem-Sabanadzovic N., Agranovsky A.A., Al Rwahnih M., Dolja V.V., Dovas C., Fuchs M., Gugerli P., Hu J.S., Jelkmann W. (2012). Taxonomic revision of the family *Closteroviridae* with special reference to grapevine leafroll-associated members of the genus *Ampelovirus* and the putative species unassigned to the family. J. Plant Pathol..

[2] Karasev A.V., Boyko V.P., Gowda S., Nikolaeva O.V., Hilf M.E., Koonin E.V., Niblett C.L., Cline K., Gumpf D.J., Lee R.F. (1995). Complete sequence of the Citrus tristeza virus RNA genome. Virology.

[3] Fraser L. (1952). Seedling yellows, an unreported virus disease of citrus. Agric. Gaz. N.S.W..

[4] Bar-Joseph M., Marcus R., Lee R.F. (1989). The continuous challenge of citrus tristeza virus control. Annu. Rev. Phytopathol..

[5] Dawson W.O., Robertson C.J., Albiach-Martí M.R., Bar-Joseph M., Garnsey S.M. (2015). Mapping sequences involved in induction of decline by citrus tristeza virus T36 on the sour orange rootstock. J. Citrus Pathol..

[6] Moreno P., Ambrós S., Albiach-Martí M.R., Guerri J., Peña L. (2008). Citrus tristeza virus: A pathogen that changed the course of the citrus industry. Mol. Plant Pathol..

[7] Fu S., Shao J., Zhou C., Hartung J.S. (2017). Co-infection of sweet orange with severe and mild strains of Citrus tristeza virus is overwhelmingly dominated by the severe strain on both the transcriptional and biological levels. Front. Plant Sci..

[8] Davino S., Willemsen A., Panno S., Davino M., Catara A., Elena S.F., Rubio L. (2013). Emergence and phylodynamics of citrus tristeza virus in Sicily, Italy. PLoS ONE.

[9] Licciardello G., Raspagliesi D., Bar-Joseph M., Catara A. (2012). Characterization of isolates of citrus tristeza virus by sequential analyses of enzyme immunoassays and capillary electrophoresis-single-strand conformation polymorphisms. J. Virol. Methods.

[10] Scuderi G., Russo M., Davino S., Ferraro R., Catara A., Licciardello G. (2016). Occurrence of the T36 Genotype of Citrus tristeza virus in Citrus Orchards in Sicily, Italy. Plant Dis..

[11] Licciardello G., Scuderi G., Ferraro R., Giampetruzzi A., Russo M., Lombardo A., Raspagliesi D., Bar-Joseph M., Catara A. (2015). Deep sequencing and analysis of small RNAs in sweet orange grafted on sour orange infected with two citrus tristeza virus isolates prevalent in Sicily. Arch. Virol..

[12] Müller G.W., Costa A.S., Price W.C. (1972). Reduction in the yield of Galego lime avoided by preimmunization with mild strains of the tristeza virus. Proceedings of the Fifth Conference of International Organization of Citrus Virologists.

[13] da Graça J.V., van Vuuren S.P., Karasev A.V., Hilf M.E. (2010). Managing Citrus tristeza virus losses using cross protection. Citrus Tristeza Virus Complex and Tristeza Diseases.

[14] Luttig M., van Vuuren S.P., van der Vyver J.B., Duran-Vila N., Milne R.G., da Graça J.V. (2002). Differentiation of single aphid cultured sub-isolates of two South African Citrus tristeza virus isolates from grapefruit by single-stranded conformation polymorphism. Proceedings of the Fifteenth Conference of International Organization of Citrus Virologists.

[15] Roistacher C.N., da Graça J.V., Müller G.W., Hilf M.E., Timmer L.W., Milne R.G., da Graça J.V. (2010). Cross protection against Citrus tristeza virus—A review. Proceedings of the Seventeenth Conference International Organization of Citrus Virologists.

[16] Ieki H., Yamaguchi A., Kano T., Koizumi M., Iwanami T. (1997). Control of stem pitting caused by Citrus tristeza virus using protective mild strains in navel orange. Ann. Phytopathol. Soc. Jpn..

[17] Lee R.F., Keremane M.L. (2013). Mild strain cross protection of tristeza: A review of research to protect against decline on sour orange in Florida. Front. Microbiol..

[18] Dawson W.O., Garnsey S.M., Tatineni S., Folimonova S.Y., Harper S.J., Gowda S. (2013). Citrus tristeza virus-host interactions. Front. Microbiol..

[19] Folimonova S.Y., Robertson C.J., Shilts T., Folimonov A.S., Hilf M.E., Garnsey S.M., Dawson W.O. (2010). Infection with strains of Citrus tristeza virus does not exclude superinfection by other strains of the virus. J. Virol..

[20] Folimonova S.Y. (2013). Developing an understanding of cross-protection by citrus tristeza virus. Front. Microbiol..

[21] Sun Y.D., Folimonova S.Y. (2019). The p33 protein of *Citrus tristeza virus* affects viral pathogenicity by modulating a host immune response. New Phytol..

[22] Folimonova S.Y. (2012). Superinfection exclusion is an active virus-controlled function that requires a specific viral protein. J. Virol..

[23] Tatineni S., French R. (2016). The coat protein and NIa protease of two Potyviridae family members independently confer superinfection exclusion. J. Virol..

[24] Harper S.J., Cowell S.J., Dawson W.O. (2017). Isolate fitness and tissue-tropism determine superinfection success. Virology..

[25] Bar-Joseph M., Catara A., Licciardello G. (2021). The puzzling phenomenon of seedling yellows recovery and natural spread of asymptomatic infections of citrus tristeza virus: Two sides of the same coin. Hortic. Rev..

[26] Albiach-Martíì M.R., Robertson C.J., Gowda S., Tatineni S., Belliure B., Garnsey S.M., Folimonova S.Y., Moreno P., Dawson W.O. (2010). The pathogenicity determinant of citrus tristeza virus causing the seedling yellows syndrome maps at the 3′-terminal region of the viral genome. Mol. Plant Pathol..

[27] Cook G., van Vuuren S.P., Breytenbach J.H.J., Steyn C., Burger J.T., Maree H.J. (2016). Characterization of Citrus tristeza virus single-variant sources in grapefruit in greenhouse and field trials. Plant Dis..

[28] Cook G., Coetzee B., Bester R., Breytenbach J.H.J., Steyn C., de Bruyn R., Burger J., Maree H. (2020). Citrus tristeza virus isolates of the same genotype differ in stem pitting severity in grapefruit. Plant Dis..

[29] Harju V.A., Skelton A., Clover G.R.G., Ratti C., Boonham N., Henry C.M., Mumford R.A. (2005). The use of real-time RT-PCR TaqMan (R) and post-ELISA virus release for the detection of beet necrotic yellow vein virus types containing RNA 5 and its comparison with conventional RT-PCR. J. Virol. Methods..

[30] Ruiz-Ruiz S., Moreno P., Guerri J., Ambrós S. (2009). Discrimination between mild and severe Citrus tristeza virus isolates with a rapid and highly specific real-time reverse transcription-polymerase chain reaction method using TaqMan LNA probes. Phytopathology..

[31] Read D.A., Pietersen G., Catara A., Bar-Joseph M., Licciardello G. (2019). Analysis of genotype composition of Citrus tristeza virus populations using Illumina Miseq technology. Methods in Molecular Biology.

[32] Okonechnikov K., Conesa A., García-Alcalde F. (2016). Qualimap 2: Advanced multi-sample quality control for high-throughput sequencing data. Bioinformatics.

[33] Harper S.J. (2013). Citrus tristeza virus: Evolution of complex and varied genotypic groups. Front. Microbiol..

[34] Tamura K., Stecher G., Kumar S. (2021). MEGA11: Molecular Evolutionary Genetics Analysis Version 11. Mol Biol Evol..

[35] Kearse M., Moir R., Wilson A., Stones-Havas S., Cheung M., Sturrock S., Buxton S., Cooper A., Markowitz S., Duran C. (2012). Geneious Basic: An integrated and extendable desktop software platform for the organization and analysis of sequence data. Bioinformatics..

[36] Mawassi M., Mietkiewska E., Gofman R., Yang G., Bar-Joseph M. (1996). Unusual sequence relationships between two isolates of Citrus tristeza virus. J. Gen. Virol..

[37] Licciardello G., Ferraro R., Scuderi G., Russo M., Catara A.F. (2021). A simulation of the use of high throughput sequencing as pre-screening assay to enhance the surveillance of citrus viruses and viroids in the EPPO Region. Agriculture.

[38] Pechinger K., Chooi K.M., MacDiarmid R.M., Harper S.J., Ziebell H. (2019). A new era for mild strain cross-protection. Viruses.

[39] Folimonova S.Y., Achor D., Bar-Joseph M. (2020). Walking together: Cross-protection, genome conservation, and the replication machinery of Citrus tristeza virus. Viruses.

[40] Butković A., González R. (2022). A brief view of factors that affect plant virus evolution. Front. Virol..

[41] Bar-Joseph M., Loebenstein G. (1973). Effects of strain, source plant, and temperature on the transmissibility of citrus tristeza virus by the melon aphid. Phytopathology.

[42] Weng Z., Liu X., Gowda S., Barthelson R.A., Galbraith D.W., Dawson W.O., Xiong Z. Extreme genome stability of citrus tristeza virus. Proceedings of the Eighteenth Conference of International Organization of Citrus Virologists.

[43] Silva G., Marques N., Nolasco G. (2012). The evolutionary rate of citrus tristeza virus ranks among the rates of the slowest RNA viruses. J. Gen. Virol..

[44] Kang S.H., Atallah O.O., Sun Y.D., Folimonova S.Y. (2018). Functional diversification upon leader protease domain duplication in the *Citrus tristeza virus* genome: Role of RNA sequences and the encoded proteins. Virology.

[45] Atallah O.O., Kang S.H., El-Mohtar C.A., Shilts T., Bergua M., Folimonova S.Y. (2016). A 5-proximal region of the Citrus tristeza virus genome encoding two leader proteases is involved in virus superinfection exclusion. Virology.

[46] Dao T.N.M., Kang S.H., Bak A., Folimonova S.Y. (2020). A non-conserved p33 protein of *Citrus tristeza virus* interacts with multiple viral partners. Mol. Plant-Microbe Interact..

[47] Bergua M., Zwart M.P., El-Mohtar C., Shilts T., Elena S.F., Folimonova S.Y. (2014). A viral protein mediates superinfection exclusion at the whole-organism level but is not required for exclusion at the cellular level. J. Virol..

[48] Sambade A., López C., Rubio L., Flores R., Guerri J., Moreno P. (2003). Polymorphism of a specific region in gene p23 of citrus tristeza virus allows discrimination between mild and severe isolates. Arch. Virol..

[49] Ruiz-Ruiz S., Navarro B., Pena L., Navarro L., Moreno P., Di Serio F., Flores R., Catara A., Bar-Joseph M., Licciardello G. (2019). Citrus tristeza virus: Host RNA silencing and virus counteraction. Methods in Molecular Biology.

[50] Nunna H., Qu F., Tatineni S. (2023). P3 and NIa-Pro of Turnip Mosaic Virus Are Independent Elicitors of Superinfection Exclusion. Viruses.

[51] Batuman O., Mawassi M., Bar-Joseph M. (2006). Transgenes consisting of a dsRNA of an RNAi suppressor plus the 3′ UTR provide resistance to Citrus tristeza virus sequences in *Nicotiana benthamiana* but not in citrus. Virus Genes.

[52] Dawson W.O., Bar-Joseph M., Garnsey S.M., Moreno P. (2015). Citrus tristeza virus: Making an ally from an enemy. Ann. Rev. Phytopath..

