# Supplementary material for: Minor Variants of Orf1a, p33, and p23 Genes of VT Strain Citrus Tristeza Virus Isolates Show Symptomless Reactions on Sour Orange and Prevent Superinfection of Severe VT Isolates
Source: Viruses. 2023 Sep 30;15(10):2037. doi: 10.3390/v15102037 (PMC10612028; doi:10.3390/v15102037)
Supplement: Supplementary file 1 [file viruses-15-02037-s001.zip › viruses-2611549-supplementary.pdf]

Supplementary Table S1. Comparative analysis of CTV detection on leaf samples taken from plant cross-protected and superinfected or not, used in the trials of CP reported in Table 2.

**CP isolate**	**SY isolate**	**Host plant**	**Inoculation**		OD 405 nm ELISA
			**CP**	**SY**	
			**Long term trials**		
**M39D**	No	SO	Oct.2014	--	1.24± 0.05
	No	SO			3.97 ± 0.25
	No	SO			2.96 ±0.04
**Mac39**	P7/4C	SO	Oct.2014	Feb.2021	0.86± 0.02
	P7/4C	SO			1.64± 0.16
	P7/4C	SO			1.61± 0.25
	P7/4C	SO			4.02± 0.16
			**Short term trials**		
**M39D**	P7/3C	SO	Jun.2020	May 2021	0.86± 0.22
	P7/3C	SO			0.49±0.07
	P7/3C	SO			0.61± 0.12
	No	SO			3.00± 0.08
	No	SO			1.32± 0.17
**M39D**	P7/3C	H SwO/SO	Jun.2021	Aug.2021	3.29±0.30
	P7/3C	H SwO/SO			3.29±0.30
	P7/3C	H SwO/SO			2.76±0.27
	P7/3C	H SwO/SO			3.14±0.60
	No	H SwO/SO			3.38±0.77
**Control**	No	H SwO/SO	--	--	0.17±0.01
		H SwO/SO			0.14±0.03
		H SwO/SO			0.16±0.01
		H SwO/SO			0.14±0.01

Leaf samples were taken two years post superinfection from plants inoculated with CP isolates in greenhouse nine or two years before and superinfected by the SY P7 isolate after even years later or two to eleven months. After superinfection plants were transferred outdoors the greenhouse. Control plants were not inoculated at all. Samples of SO inoculated only with SY isolates were not available for testing because the plants declined and died about one year post inoculation. Reading. Each value is a mean of the absorbance values of three technical replicates measured at 405 nm after 30 min of incubation at room temperature.
